# Time-dependent improvement of quality of life with teriparatide or alendronate therapy: a JOINT-05 sub-analysis

**DOI:** 10.1007/s00774-025-01621-y

**Published:** 2025-07-31

**Authors:** Satoshi Mori, Shiro Tanaka, Tatsuhiko Kuroda, Hiroshi Hagino, Satoshi Soen

**Affiliations:** 1 Department of Bone and Joint Surgery, Seirei Hamamatsu General Hospital, 2-12-12 Sumiyoshi, Hamamatsu, Shizuoka 430-8558 Japan; 2 Department of Clinical Biostatistics, Graduate School of Medicine, Kyoto University, Kyoto, Japan; 3 Public Health Research Foundation, Shinjuku-Ku, Tokyo, Japan; 4 Department of Rehabilitation, Sanin Rosai Hospital, Yonago, Tottori Japan; 5 Osteoporosis and Rheumatology Clinic, Soen Orthopaedics, Kobe, Hyogo Japan

**Keywords:** Alendronate, Fracture risk, Osteoporosis, Teriparatide, Quality of life

## Abstract

**Introduction:**

The effects of teriparatide (TPTD) and alendronate (ALN) on health-related quality of life (HRQOL) were evaluated in osteoporotic patients with high fracture risk using data from the JOINT-05 trial.

**Materials and methods:**

JOINT-05 was a randomized, controlled trial comparing the fracture prevention effects of sequential therapy with TPTD for 72 weeks followed by ALN for 48 weeks with those of ALN monotherapy for 120 weeks. This sub-analysis evaluated the effects of TPTD and ALN on HRQOL using data from baseline to 72 weeks. HRQOL was assessed at baseline, 4, 12, 24, 48, and 72 weeks using the EuroQoL-5Dimension (EQ-5D) questionnaire containing five domains (mobility, self-care, usual activities, pain/discomfort, and anxiety/depression). Scores were also transformed using utility weights derived from the general Japanese population.

**Results:**

This sub-analysis included 476 patients in the TPTD group and 492 patients in the ALN group. No significant differences in utility scores were observed between groups at all times. Utility scores improved significantly after 12 weeks in the TPTD group and after 24 weeks in the ALN group. Mobility and self-care scores improved significantly in the TPTD group. Significant improvements in usual activity, pain/discomfort, and anxiety/depression scores occurred earlier in the TPTD group.

**Conclusion:**

Although there were no significant differences in utility scores between the TPTD and ALN groups, significant improvements in the utility score and 5 domain scores were observed earlier in the TPTD group.

When treating osteoporosis patients with a high risk of fracture, the selecting treatment drugs may be decided with primary consideration of its effect on fracture prevention, followed by its effect on improving HRQOL.

## Introduction

Osteoporosis is defined as a systemic skeletal disease characterized by low bone mass and microarchitectural deterioration of bone tissue with a consequent increase in bone fragility and susceptibility to fracture [1].

Fragility fractures in patients with osteoporosis have been associated with increased functional impairment, reduced health-related quality of life (HRQOL), and increased risk of mortality [2–4]. Apart from this relationship, bone mineral density (BMD) was positively associated with HRQOL in postmenopausal women [5].

Thus, it is important to assess fracture risk at an early stage and then to start the necessary treatment to increase BMD and prevent incident fractures to manage HRQOL [6].

Regarding bone resorption inhibitors (bisphosphonates), large-scale studies have compared their effects on HRQOL with those of placebo, but their results have been inconsistent. It has been reported that utility scores measured by EuroQoL-5 Dimension (EQ-5D) were comparable for zoledronic acid and placebo (HORIZON trial); though more patients given placebo consistently had extreme difficulty in mobility, self-care, and usual activities, no significant differences were observed [7]. In comparisons between bisphosphonates, there were no significant differences in QOL scores between patients receiving zoledronic acid or alendronate for 12 months [8].

For teriparatide (TPTD), a bone anabolic agent, several single-arm observational studies have reported its administration and changes in HRQOL [9–11]. In these studies, significant improvements in HRQOL compared with baseline were observed. In comparable studies, TPTD showed significant improvement compared with supplemental agents (calcium plus vitamin D treatment) for 24 months [12]. Panico et al. investigated changes in HRQOL when TPTD or alendronate (ALN) was administered in a randomized, prospective, cohort study [13]. They reported that each treatment group showed significant improvements in daily living, performance of domestic jobs, and locomotor function, but the assessments were only conducted at baseline and at 18 months. None of the above reports evaluated the effect of TPTD on HRQOL continuously from the start of treatment using an active drug as a control.

In this sub-analysis, data from JOINT-05 [14–16], which was a randomized, controlled trial (RCT) involving postmenopausal Japanese women at high risk of fracture comparing the efficacy of fracture prevention between a sequential TPTD followed by ALN group and an ALN monotherapy group for 120 weeks, were used to elucidate the time-dependent change of HRQOL and the difference between the drug regimens.

## Materials and methods

### Patients and measurements

JOINT-05 enrolled Japanese women aged 75 years or older with primary osteoporosis (Trial registration numbers: jRCTs031180235 and UMIN000015573) [14]. The patients were randomly assigned (1:1) to receive sequential therapy with TPTD for 72 weeks followed by ALN for 48 weeks or monotherapy with ALN for 120 weeks. TPTD 56.5 μg was injected once weekly, and ALN was provided as a 5-mg tablet (once daily), 35-mg tablet or jelly (once weekly), or 900-μg infusion (once every 4 weeks). Native vitamin D 400 supplements were provided to both groups throughout the entire treatment period.

Patients at high risk of fracture, defined by lower bone mineral density (BMD) and higher number or grade of prevalent vertebral fractures, and ambulatory patients who could walk independently were included. The detailed inclusion criteria have been described elsewhere [14]. JOINT-05 study indicated that the incidence rate of morphometric vertebral fractures was lower with treatment using once-weekly TPTD followed by ALN, compared with treatment with ALN alone throughout the study [15, 16]. The protocol was conducted according to the Declaration of Helsinki. Written, informed consent was obtained from all patients before they underwent any examinations. This study was approved by the central and onsite institutional review boards (IRBs).

To compare the drug efficacy for HRQOL between TPTD and ALN treatment, the full analysis dataset of JOINT-05 [15] up to 72 weeks was analyzed.

### Outcome measures

HRQOL was assessed by patients at baseline (0 weeks) and 4, 12, 24, 48, and 72 weeks using the EuroQoL-5 Dimension (EQ-5D) questionnaire [17]. Patients rated their current health state in five domains (mobility, self-care, usual activities, pain/discomfort, and anxiety/depression), scoring each domain on a 3-point severity level (1: no problem, 2: some or moderate problems, or 3: extreme problems). The scores were also transformed using utility weights derived from the general Japanese population (ranging from −1 to 1). Higher scores indicate better overall health status.

### Statistical analysis

Baseline numerical data are presented as mean ± standard deviation values, whereas categorical data are expressed as numbers and proportions (%). Utility scores and the 5 domain scores are reported as mean and 95% confidence intervals (CI) values.

The utility score and scores of each domain of EQ-5D from baseline to 72 weeks were analyzed using linear mixed models for repeated measures. The models included random effects for patients and fixed effects of treatment, visit (as categorical), and treatment-by-visit interactions. The correlation structure was assumed to be compound symmetry. To describe the trend of EQ-5D over time, LS means from baseline to 72 weeks and their 95% CIs were estimated using the linear mixed models. Contrast tests were also performed based on the regression coefficients for four contrasts representing whether there was a difference between the two groups for each visit and eight contrasts representing whether there was a difference from baseline for each of the two groups. Because HRQOL was a secondary endpoint of JOINT-05, and these tests were exploratory in nature, p-values without adjustment for multiplicity were reported. It was assumed that missing data were missing at random, and likelihood-based ignorable analysis was performed, that is, all available data were analyzed without imputing missing data for EQ-5D scores. All data were analyzed with SAS software version 9.4 (SAS Institute, Cary, NC). *P* values < 0.05 were considered significant.

## Results

The full analysis dataset of the original JOINT-05 consisted of the TPTD-ALN group (*N* = 489) and the ALN group (*N* = 496). Their baseline characteristics are shown in Table 1. There were no significant differences between the groups. This sub-analysis for evaluating HRQOL included 476 patients in the TPTD-ALN group and 492 patients in the ALN group.

**Table 1:** Baseline characteristics

	TPTD group (*N* = 489)	ALN group (*N* = 496)
Age, y	81.4 ± 4.5	81.5 ± 4.7
Age at menopause, y	49.6 ± 4.4	49.2 ± 4.4
BMI, kg/m^2^	22.2 ± 3.8	22.1 ± 3.5
Number of prevalent vertebral fractures	1.7 ± 1.9	1.8 ± 2.0
0	32.3%	32.1%
1	27.0%	27.2%
2	16.8%	15.1%
3	10.6%	9.5%
4	4.9%	6.0%
≥ 5	8.4%	10.1%
Maximum grade of prevalent vertebral fractures		
Grade 1	9.2%	9.5%
Grade 2	15.7%	17.7%
Grade 3	42.7%	40.7%
History of proximal femoral fractures, yes, %	14.1%	13.5%
L2-4 BMD, T-score	−2.3 ± 1.4	−2.4 ± 1.4
Prior treatment, yes, %	53.8%	54.4%
Prior bisphosphonates, yes, %	29.7%	30.2%
EQ-5D mobility	1.5 ± 0.5	1.5 ± 0.5
Self-care	1.3 ± 0.5	1.3 ± 0.5
Usual activities	1.5 ± 0.5	1.5 ± 0.5
Pain/discomfort	1.7 ± 0.6	1.7 ± 0.6
Anxiety/depression	1.3 ± 0.5	1.3 ± 0.5

Values are indicated as means ± SD or percentages

*TPTD*: teriparatide, *ALN*: alendronate, *BMI*: body mass index, *BMD*: bone mineral density

**Fig. 1:**
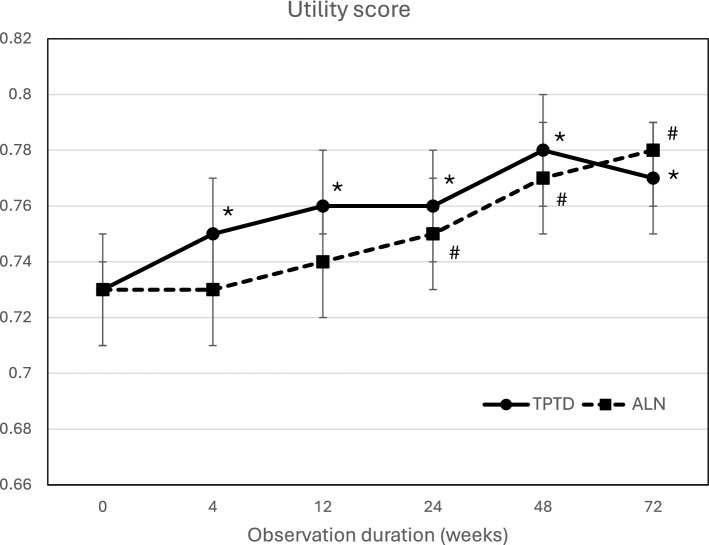
Utility score of EQ-5D at each measurement point by treatment group. Values shown are means and 95% CIs. *TPTD* Teriparatide, *ALN* Alendronate. *: *p* < 0.05, versus baseline in the TPTD group; #: *p* < 0.05, versus baseline in the ALN group

The mean and 95% CI of the utility score of EQ-5D at each measurement point by treatment group are shown in Fig. 1. No significant differences were observed between the TPTD group and the ALN group at all measurement points. Change from baseline in the utility score was significantly improved after 12 weeks in the TPTD group and after 24 weeks in the ALN group, and the effects were sustained thereafter (*p* < 0.05).

**Fig. 2:**
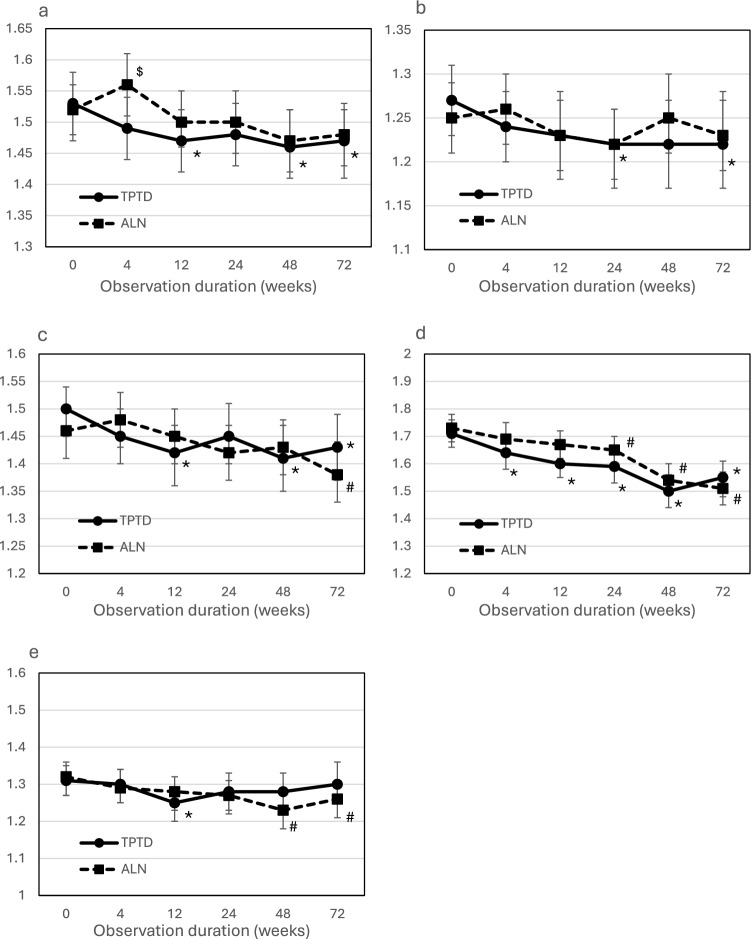
Differences at each measurement point between the treatment groups and change from baseline in the EQ-5D score in the **a** mobility, **b** self-care, **c** usual activities, **d** pain/discomfort, and **e** anxiety/depression domains**.** Values shown are means and 95% Cis. *TPTD* Teriparatide, *ALN* Alendronate. ^$^: *p* < 0.05, between the TPTD and ALN groups; *: *p* < 0.05, versus baseline in the TPTD group; ^#^: *p* < 0.05, versus baseline in the ALN group

Differences at each measurement point between the treatment groups and change from baseline in the EQ-5D score in 5 domains (mobility, self-care, usual activities, pain/discomfort, and anxiety/depression) are shown in Fig. 2.

Significant differences were not observed between the TPTD and ALN groups for all domains of EQ-5D at each measurement point, except for the mobility score at 4 weeks. The mobility score was significantly improved at 12, 48, and 72 weeks in the TPTD group, and no significant change was observed in ALN group. The self-care score was significantly improved at 24 and 72 weeks in the TPTD group. The usual activity score was significantly improved at 12, 48, and 72 weeks in the TPTD group and at 72 weeks in the ALN group. In the pain/discomfort domain, significant improvement was observed after 12 weeks in the TPTD group and after 24 weeks in the ALN group. The anxiety/depression score was significantly improved at 12 weeks in the TPTD group and after 48 weeks in the ALN group.

## Discussion

A sub-analysis of data from baseline to 72 weeks from the previously conducted RCT JOINT-05 was performed, comparing the effects of TPTD and ALN on HRQOL. Although there were no differences between the treatment arms, several significant differences were observed in changes from baseline.

The utility score of the EQ-5D, scores of self-care, usual activity, pain/discomfort, and anxiety/depression of the five domains of HRQOL showed earlier improvement in the TPTD group.

Several hypotheses can be considered regarding the mechanism behind these differences.

In the original JOINT-05 study, the incidence of morphometric vertebral fracture was significantly lower in the TPTD group than in the ALN group over the 72-week observation period [15]. Indeed, previous reports have shown that vertebral fractures have a negative impact on HRQOL [18]. It is assumed that vertebral fractures cause deformation of the trunk and limit range of motion, which restricts physical movement, activity, or psychological stress, resulting in a decrease in patients’ HRQOL.

From another perspective, Miyamura et al. reported that treatment with PTH prevented bone loss, mechanical hyperalgesia, and osteocyte increase. The inhibitory effect of PTH on osteoclasts might contribute to the improvement of skeletal pain [19]. Moreover, it was reported that TPTD treatment improved lung function and decreased pain intensity in women with multiple osteoporotic vertebral compression fractures [20]. Thus, these mechanisms of TPTD may be responsible for the difference in HRQOL compared with ALN.

Regarding the effect on fracture healing of TPTD, several meta-analyses and systematic reviews have been reported, but the results are inconsistent. A meta-analysis showed that administration of TPTD following a fracture lacked effectiveness for fracture healing [21]. Another review reported that TPTD treatment might benefit fracture healing, lowering the rate of delayed union and nonunion and shortening fracture healing time [22, 23]. Therefore, it cannot be concluded that the difference in efficacy observed in the present analysis is due to promotion of fracture healing by TPTD treatment.

The ALN treatment group also showed significant improvement in domains of usual activity, pain/discomfort, and anxiety/depression in the EQ-5D at the later stages of treatment compared with baseline.

It has been reported that the mechanism by which ALN improves HRQOL is by improving pain associated with osteoporosis through inhibition of TRPV1 [24], and, furthermore, an RCT showed that ALN has a stronger analgesic effect than calcitonin in women with postmenopausal osteoporosis [25].

There is a limitation in this study. This sub-analysis was based on data from patients enrolled in an RCT (JOINT-05 [14, 15]) to evaluate the efficacy and safety of an anabolic agent (TPTD). The inclusion criteria for the original study specified Japanese osteoporotic women aged 75 years or older with a high risk of fracture and did not include lower fracture risk patients. However, ALN, which was used as a control drug, is used to treat younger or lower fracture risk patients. Therefore, further studies including younger age groups and patients with a lower fracture risk may be needed to confirm the improvement in HRQOL with ALN.

In conclusion Although there were no differences in the EQ-5d utility score between the TPTD and ALN groups, some significant changes from baseline were observed in five domain scores, with faster improvement observed in the TPTD group.

When treating osteoporosis patients with a high risk of fracture, the selecting treatment drugs may be decided with primary consideration of its effect on fracture prevention, followed by its effect on improving HRQOL.

## Data Availability

The data that support the findings of this study are available from the corresponding author upon reasonable request.
