# Correction: Time-dependent improvement of quality of life with teriparatide or alendronate therapy: a JOINT-05 sub-analysis

**DOI:** 10.1007/s00774-025-01637-4

**Published:** 2026-02-13

**Authors:** Satoshi Mori, Shiro Tanaka, Tatsuhiko Kuroda, Hiroshi Hagino, Satoshi Soen

**Affiliations:** 1 Department of Bone and Joint Surgery, Seirei Hamamatsu General Hospital, 2-12-12 Sumiyoshi, Hamamatsu, Shizuoka 430-8558 Japan; 2 Department of Clinical Biostatistics, Graduate School of Medicine, Kyoto University, Kyoto, Japan; 3 Public Health Research Foundation, Shinjuku-Ku, Tokyo, Japan; 4 Department of Rehabilitation, Sanin Rosai Hospital, Yonago, Tottori Japan; 5 Osteoporosis and Rheumatology Clinic, Soen Orthopaedics, Kobe, Hyogo Japan

**Correction: Journal of Bone and Mineral Metabolism (2025)** 10.1007/s00774-025-01621-y

The authors identified two errors in the original article. The significant change in utility score from the start of treatment in the TPTD group was observed from week 4, not week 12 (Fig. 1). The following sentence in the Abstract, “Utility scores improved significantly after 12 weeks in the TPTD group and after 24 weeks in the ALN group.” was incorrect. This should have read as follows: “Utility scores improved significantly after 4 weeks in the TPTD group and after 24 weeks in the ALN group.” Similarly, the following sentence in the Results was incorrect: “Change from baseline in the utility score was significantly improved after 12 weeks in the TPTD group and after 24 weeks in the ALN group, and the effects were sustained thereafter (*p* < 0.05).” The correct sentence was as follows: “Change from baseline in the utility score was significantly improved after 4 weeks in the TPTD group and after 24 weeks in the ALN group, and the effects were sustained thereafter (*p* < 0.05).”

